# Dietary advanced glycation end-products and their associations with body weight on a Mediterranean diet and low-fat vegan diet: a randomized, cross-over trial

**DOI:** 10.3389/fnut.2024.1426642

**Published:** 2024-08-08

**Authors:** Hana Kahleova, Tatiana Znayenko-Miller, Giulianna Motoa, Emma Eng, Alex Prevost, Jaime Uribarri, Richard Holubkov, Neal D. Barnard

**Affiliations:** ^1^Physicians Committee for Responsible Medicine, Washington, DC, United States; ^2^Charles E. Schmidt College of Medicine, Florida Atlantic University, Boca Raton, FL, United States; ^3^Icahn School of Medicine at Mount Sinai, New York, NY, United States; ^4^School of Medicine, University of Utah, Salt Lake City, UT, United States; ^5^Adjunct Faculty, George Washington University School of Medicine and Health Sciences, Washington, DC, United States

**Keywords:** advanced glycation end-products, diet, Mediterranean, nutrition, vegan, weight

## Abstract

**Objective:**

Evidence suggests that changes in dietary advanced glycation end-products (AGEs) may influence body weight, but the effects of different dietary patterns remain to be explored.

The aim of this study was to compare the effects of a Mediterranean and a low-fat vegan diet on dietary AGEs and test their association with body weight.

**Materials and methods:**

In this randomized cross-over trial, 62 overweight adults were assigned to a Mediterranean or a low-fat vegan diet for 16-week periods in random order, separated by a 4-week washout. Body weight was the primary outcome. Three-day diet records were analyzed using the Nutrition Data System for Research software and dietary AGEs were estimated, using an established database. Statistical approaches appropriate for crossover trials were implemented.

**Results:**

Dietary AGEs decreased by 73%, that is, by 9,413 kilounits AGE/day (95% −10,869 to −7,957); *p* < 0.001, compared with no change on the Mediterranean diet (treatment effect −10,303 kilounits AGE/day [95% CI −13,090 to −7,516]; *p* < 0.001). The participants lost 6.0 kg on average on the vegan diet, compared with no change on the Mediterranean diet (treatment effect −6.0 kg [95% CI −7.5 to −4.5]; *p* < 0.001). Changes in dietary AGEs correlated with changes in body weight (*r* = +0.47; *p* < 0.001) and remained significant after adjustment for total energy intake (*r* = +0.39; *p* = 0.003).

**Conclusion:**

Dietary AGEs did not change on the Mediterranean diet but decreased on a low-fat vegan diet, and this decrease was associated with changes in body weight, independent of energy intake.

**Clinical trial registration:**

https://clinicaltrials.gov/, identifier NCT03698955

## Introduction

Approximately 70% of US adults are overweight (1), and the extra weight is often associated with an increased cardiometabolic risk (2), partly via an accelerated production of advanced glycation end products (AGEs). AGEs are a large heterogenous group of compounds formed during a non-enzymatic reaction of the carbonyl groups of sugars with free amino groups in protein (3). They cause inflammation and oxidative stress, and thus accelerate the development of chronic diseases, particularly type 2 diabetes and cardiovascular disease. While AGEs are generated constantly during metabolism, they are also ingested through the diet (4, 5). Several randomized interventional trials have shown that dietary AGE restriction ameliorates insulin resistance in obese people with metabolic syndrome (6, 7).

Thermally prepared foods, particularly of animal origin, are rich in AGEs, which may be very flavorful, and therefore enhancing their palatability and consumption, thus promoting weight gain. Databases with the AGE content of different food items have been published in the literature and provide the basis to estimate AGE content in consumed foods, as well as the design of low-AGE diets (5, 8). We have extensively used one of these databases (5) including ELISA measurement of carboxymethyllysin (CML) and shown an association between dietary AGE intake and circulating AGE levels, as well as markers of oxidative stress, inflammation, innate defenses and insulin resistance in different populations (5–7).

Mediterranean and vegan diets have been recognized as healthy dietary patterns that may be beneficial for weight loss and cardiometabolic health, but the extent to which these dietary patterns affect AGE ingestion and the resulting associations between AGEs and body weight still need to be explored.

The aim of this secondary analysis of a randomized crossover trial, which compared a Mediterranean and low-fat vegan diet head-to-head in overweight adults and showed a greater weight loss and improvements in cardiometabolic outcomes on the low-fat vegan diet (9), was to compare the effects of these two diets on dietary AGEs and test their association with body weight. We hypothesized that a low-fat vegan diet would result in a reduction of dietary AGEs, compared with the Mediterranean diet, and that these changes would be associated with changes in body weight.

## Materials and methods

### Study design and eligibility

The methods of the overall study have been described in detail previously (9). Briefly: This randomized, cross-over trial took place between February and October 2019 in Washington, DC. We enrolled adults, aged 30–76 years, with a body mass index between 28 and 40 kg/m^2^. Exclusion criteria included type 1 diabetes, smoking, pregnancy or lactation, alcohol or drug abuse, or already following a vegan or Mediterranean diet. The study was conducted in accordance with the Declaration of Helsinki, and the study protocol was approved by the Advarra Institutional Review Board, located in Columbia, MD, USA, on September 20, 2018 (protocol identification number Pro00029777). The study was registered on ClinicalTrials.gov (ID: NCT03698955). All participants gave informed, written consent.

### Randomization and study groups

Participants were randomized in a 1:1 ratio into 2 groups. Group 1 started with a Mediterranean diet for 16 weeks, followed by a 4-week wash-out period, and then switched to a low-fat vegan diet for 16 weeks. Group 2 followed a low-fat vegan diet for 16 weeks, and after a 4-week wash-out period, they adopted the Mediterranean diet for 16 weeks. Participants were assessed at weeks 0, 16, 20, and 36.

The Mediterranean diet was based on the PREDIMED protocol (10), which includes ≥2 servings/day of vegetables, ≥2–3 servings/day of fresh fruits, ≥3 servings/week of legumes, ≥3 servings/week of fish or shellfish, and ≥3 servings/week of nuts or seeds, and favors lean white meats over red meats. Participants were discouraged from consuming cream, butter, margarine, processed meats, sweetened beverages, pastries, and processed snacks. Participants were instructed to use the provided extra virgin olive oil (50 g daily) as their main culinary fat.

The low-fat vegan diet consisted of fruits, vegetables, grains, and legumes. It was an *ad libitum* diet, with no instructions on portion sizes. Animal products and added fats were excluded and vitamin B_12_ was supplemented (a supplement with 500 μg/day was provided). Both diets were *ad libitum* diets, with no meals provided for either intervention. Alcohol was limited to one beverage/day for women, and two beverages/day for men. Participants were instructed to keep their physical activity and prescribed medications constant, unless otherwise directed by their personal physicians. Participants attended weekly classes specific to their assigned diets for the whole intervention period. These classes provided nutrition education, recipes, meal plans, as well as group support.

Body weight was measured, using an electronic scale accurate to 0.1 kg. Body composition was measured by dual energy x-ray absorptiometry (Lunar iDXA, GE Healthcare; Madison, WI) with Encore^®^ 2005 v.9.15.010 software, equipped with the CoreScan module (GE Healthcare, Madison, WI) to measure visceral adipose tissue volume. Diet adherence was checked weekly, using short questionnaires that captured all important components of each diet. Participants submitted a 3-day diet record at weeks 0, 16, 20, and 36. Dietary data were reviewed and analyzed by a Registered Dietitian or a team member certified in Nutrition Data System for Research version 2018 (Nutrition Coordinating Center, University of Minnesota, Minneapolis, MN) (11).

In a post-hoc analysis of above data, AGE scores were assigned to each food item, using a published database of AGE content that has been previously used in epidemiologic studies to estimate dietary AGE intake (12–14). Consistent with previously published methodology (12–14), each food item identified was assigned a dietary AGE value in kilounits AGE/gram of food, which was then multiplied by the number of grams of this food consumed per day. The dietary AGE values for all foods consumed during the day were then summed to provide a total dietary AGE value in kilounits AGE/day per participant. The database used contains a large number of food items, the AGE content of which is expressed as kilounits AGE/gram of food (5). Whenever a food present in the Nutrition Data System for Research was not listed in the dietary AGE database, a value was assigned based on the similarity of nutrient ingredients and cooking methods with foods listed in the dietary AGE database. This was done by a co-author (JU) who was masked regarding dietary intervention assignment. Less than 10% of food items in the current cohort was estimated this way and the estimation included in the calculation of the total dietary AGE intake. In the database, carboxymethyllysin-AGE content was estimated using ELISA based on monoclonal anti- carboxymethyllysin antibody (5).

### Statistical analysis

Power analysis: Assuming a 70% reduction on the vegan diet, which is a conservative estimate based on our previous publication (15), and a 10% reduction on the Mediterranean diet, with a standard deviation of 9,800 kilounits AGE/day (ku/day), the power to detect the 8,000-point difference is 99.99% with 62 participants, and 99.98% with the 50 participants who completed the entire crossover study.

The statistical analysis was performed in all participants with complete data across all timepoints. Treatment effect, under the assumption of no carryover effect in a crossover trial, was quantified by comparing changes from baseline (from week 0 to week 16, and from week 20 to week 36), within study participants while on Mediterranean versus vegan diet, using paired t-tests (an approach yielding estimates and significance levels identical to a mixed model analysis controlling for participant). Thus, the reported treatment effect is the mean difference between each participant’s outcomes on the vegan versus the Mediterranean diet. Carryover effect was then assessed by comparing treatment effects by the first diet that each participant received using two-sample t-tests (an approach equivalent to testing for an interaction between treatment and first diet in an analysis of variance model). Because of a detected carry-over effect (difference in magnitude of treatment effect according to the first diet each participant received) for several outcomes including total AGEs, period-specific estimates of treatment effects were also determined for all outcomes. These treatment effects were compared by (within-period) diet using two-sample t-tests. Within each intervention, paired comparison t-tests were also calculated to test whether the changes from baseline to 16 weeks in each treatment period were statistically significant. The statistician was blinded to dietary interventions. Results are presented as means with 95% confidence intervals (CI); formal correction for multiple comparisons was not performed as this study of secondary outcomes is viewed as hypothesis-generating.

## Results

### Participant characteristics

Of 506 people screened by telephone, 62 met participation criteria and were randomly assigned to start with the Mediterranean (*n* = 32) or the vegan diet (*n* = 30) diet (Supplementary Figure 1 and Supplementary Table 1).

### Dietary AGEs

**FIGURE 1:**
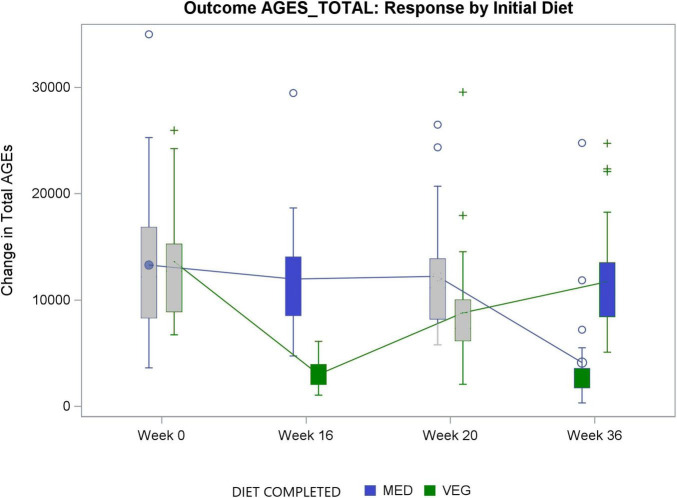
Total AGEs and their changes in response to both diets. MED, The Mediterranean diet; VEG, The low-fat vegan diet.

Dietary AGEs decreased by 73% on the low-fat vegan diet, that is by 9,413 kilounits AGE/day (95% −10,869 to −7,957); *p* < 0.001, compared with no change on the Mediterranean diet (treatment effect −10,303 kilounits AGE/day [95% CI −13,090 to −7,516]; *p* < 0.001). The reduction of dietary AGEs on the low-fat vegan diet came mainly from excluding the consumption of meat (41%), and further from minimizing the consumption of added fats (27%) and avoiding dairy products (14%; see Table 1). The changes in dietary AGEs in response to both diets during the whole study are shown in Figure 1 and Table 2. There was a statistically significant carryover effect for total AGEs (*p* = 0.01), and therefore an additional ANOVA for repeated measures was performed, conservatively assessing treatment effect from the first study period alone (−9,578 ku/day [95% CI −13,014 to −6,143]; *p* < 0.001; Table 3).

**TABLE 1:** Changes in dietary Advanced Glycation End-Products (AGEs) during the study comparing a Mediterranean and low-fat vegan diet.

Variable	Mediterranean Baseline	Mediterranean Final	Δ Mediterranean	Vegan Baseline	Vegan Final	Δ Vegan	Treatment Effect	*P*-value
**Dietary AGEs (ku/day)**								
Total AGEs (ku/day)	10,943 (9,083 to 12,803)	11,833 (10,361 to 13,305)	890 (−1,451 to 3,231)	12,939 (11,428 to 14,450)	3,526 (2,503 to 4,549)	−9,413 (−10,869 to −7,957)***	−10,303 (−13,090 to −7,516)	<0.001
Fruit	203 (93 to 313)	125 (53 to 196)	−79 (−180 to 23)	185 (82 to 287)	34 (3–66)	−150 (−259 to −42)**	−72 (−181 to 38)	0.20
Vegetables	199 (152–245)	217 (179–254)	18 (−47 to 83)	164 (118–210)	278 (234–323)	114 (58 to 170)***	96 (8 to 184)	0.03
Grains	821 (599 to 1042)	582 (470 to 693)	−239 (−498 to 21)	844 (652 to 1036)	729 (587 to 872)	−115 (−325 to 95)	124 (−211 to 459)	0.46
-Whole grains	171 (129 to 214)	240 (180 to 300)	69 (0.4 to 137)*	202 (138 to 266)	233 (183 to 282)	31 (−47 to 108)	−38 (−140 to 64)	0.46
-Some whole grains	71 (22 to 119)	48 (11 to 84)	−23 (−82 to 36)	59 (18 to 101)	85 (20 to 150)	25 (−55 to 105)	48 (−51 to 147)	0.33
-Refined grains	579 (370 to 787)	294 (189 to 399)	−285 (−530 to −39)*	583 (429 to 737)	412 (273 to 551)	−171 (−384 to 42)	114 (−216 to 443)	0.49
Meat	3616 (2623 to 4609)	2939 (2101 to 3778)	−677 (−1963 to 609)	5182 (4071 to 6292)	285 (−223 to 793)	−4,896 (−5,841 to −3,952)***	−4,219 (−5,915 to −2,523)	<0.001
-Red meat	971 (516 to 1,425)	192 (18 to 366)	−779 (−1,252 to −306)**	1147 (667 to 1,626)	120 (−99 to 339)	−1,027 (−1,500 to −554)***	−248 (−903 to 406)	0.45
-White meat	2,439 (1,660 to 3,217)	2,575 (1,737 to 3,413)	137 (−1,023 to 1,296)	3,642 (2,721 to 4,562)	138 (−99 to 375)	−3,504 (−4,322 to −2,685)***	−3,641 (−5,119 to −2,162)	<0.001
-Fried meat	212 (0 to 428)	153 (0 to 314)	−58.4 (−338 to 221)	652 (59 to 1,245)	0	−652 (−1,245 to −59)*	−594 (−1,254 to 67)	0.08
-Processed meat	207 (102 to 313)	172 (73 to 271)	−35 (−184 to 113)	393 (244 to 542)	27 (−28 to 82)	−366 (−530 to −201)***	−330 (−534 to −127)	0
-Seafood	999 (645 to 1,353)	1,457 (1,129 to 1,784)	457 (97 to 818)*	722 (439 to 1,005)	10 (−1 to 22)	−711 (−995 to −428)***	−1,169 (−1,627 to −710)	<0.001
Eggs	378 (171 to 584)	492 (298 to 686)	114 (−161 to 390)	530 (317 to 742)	1 (0 to 2)	−528 (−741 to −315)***	−643 (−958 to −327)	0
-Baked	9 (5 to 14)	4 (2 to 6)	−5 (−10 to −0)*	8 (4 to 12)	1 (0 to 2)	−7 (−11 to −3)**	−1 (−7 to 5)	0.64
-Boiled	1 (0 to 3)	2 (0 to 4)	1 (−2 to 4)	1 (−1 to 2)	0	−1 (−2 to 1)	−1 (−4 to 2)	0.38
-Fried	367 (162 to 572)	486 (292 to 680)	119 (−156 to 394)	521 (308 to 734)	0	−521 (−734 to −308)***	−640 (−954 to −326)	0
Nuts & Seeds	688 (344 to 1,032)	1,182 (846 to 1,519)	494 (106 to 883)*	721 (425 to 1,016)	296 (165 to 426)	−425 (−745 to −106)*	−919 (−1,462 to −377)	0
Meat alternatives	132 (39 to 225)	198 (27 to 369)	66 (−129 to 262)	116 (−16 to 247)	593 (347 to 840)	478 (285 to 670)***	411 (171 to 651)	0
Dairy	1,895 (921 to 2,869)	961 (578 to 1,343)	−935 (−1,960 to 91)	2,519 (1,757 to 3,281)	193 (−32 to 417)	−2,327 (−3,128 to −1,526)***	−1,392 (−2,572 to −212)	0.02
-Full-fat	1,113 (357 to 1,869)	718 (394 to 1,042)	−395 (−1,189 to 399)	1,340 (622 to 2,058)	168 (−50 to 386)	−1,172 (−1,940 to −404)**	−777 (−2,002 to 448)	0.21
-Reduced fat	183 (25 to 342)	234 (65 to 403)	51 (−190 to 292)	246 (113 to 378)	24 (−24 to 73)	−221 (−354 to −88)*	−272 (−584 to 40)	0.09
-Non-fat	599 (30 to 1,167)	9 (−8 to 25)	−590 (1,160 to −21)*	934 (424 to 1,443)	0	−933 (−1,443 to −424)***	−343 (−1,167 to 481)	0.41
Dairy alternatives	94 (−4 to 191)	4 (0 to 8)	−89 (−187 to 8)	237 (70 to 404)	10 (5 to 15)	−228 (−395 to −60)**	−138 (−341 to 65)	0.18
Added fat	1,659 (1,242 to 2,076)	3,375 (2,838 to 3,912)	1,716 (1,119 to 2,314)***	1,504 (1,205 to 1,803)	433 (278 to 587)	−1,071 (−1,437 to −706)***	−2,788 (−3,498 to −2,077)	<0.001
-Animal	708 (391 to 1,025)	229 (116 to 342)	−479 (−822 to −136)**	693 (477 to 910)	85 (−3 to 174)	−608 (−850 to −366)***	−129 (−512 to 254)	0.50
-Vegetable	951 (632 to 1,271)	3,146 (2,581 to 3,712)	2,195 (1,635 to 2,755)***	811 (579 to 1,043)	348 (241 to 454)	−463 (−727 to −200)***	−2,658 (−3,359 to −1,958)	<0.001
Added sugar	91 (52 to 130)	72 (12 to 131)	−19 (−89 to 51)	63 (23 to 102)	33 (−2 to 69)	−29 (−85 to 27)	−10 (−96 to 76)	0.82
Sugar-sweetened beverages	2 (1 to 3)	1 (0 to 1)	−1 (−2 to 0)*	2 (1 to 3)	1 (0 to 1)	−1 (−2 to −1)*	0 (−1 to 1)	0.53
Alcohol	6 (3 to 10)	8 (4 to 11)	1 (−3 to 6)	9 (5 to 13)	5 (2 to 8)	−4 (−7 to 0)*	−5 (−10 to 0)	0.03

Data are means and estimated treatment effects with 95% confidence intervals. P values for treatment effect are from paired *t*-tests comparing mean changes within each participant while on each treatment arm.

*p < 0.05,

**p < 0.01 and

***p < 0.001 for within-group changes from baseline assessed by paired *t*-tests.

**TABLE 2:** Changes in dietary Advanced Glycation End-Products (AGEs) and estimated treatment effects for the first and the second period of the study, comparing a Mediterranean and a low-fat vegan diet.

AGEs (ku/day)	ΔMediterranean (Participants with First Diet Mediterranean)	ΔVegan (Participants with First Diet Mediterranean)	Treatment Effect: Participants whose First Diet was Mediterranean	*P*-value for Participants whose First Diet was Mediterranean	ΔMediterranean (Participants with First Diet Vegan)	ΔVegan (Participants with First Diet Vegan)	Treatment Effect: Participants whose First Diet was Vegan	*P*-value for Participants whose First Diet was Vegan	*P*-value: test for carryover effect
**Dietary AGEs (ku/day)**									
Total AGEs (ku/day)	−1,314 (−5,017 to +2,389)	−8,110 (−12,625 to −8,606)***	−6,796 (−10,819 to −2,773)	0.002	+2,925 (−23 to +5,873)	−10,616 (−12,625 to −8,606)***	−13,541 (−17,204 to −9,877)	<0.001	0.0135
Fruit	−53 (−196 to +90)	−85 (−239 to +69)	−32 (−223 to 159)	0.73	−103 (−256 to +51)	−211 (−370 to −52)*	−108 (−237 to +20)	0.095	0.49
Vegetables	+42 (−38 to +121)	+165 (+110 to +219)***	+123 (+10 to +237)	0.04	−4 (−109 to +102)	+68 (−28 to +164)	+71 (−69 to +212)	0.30	0.56
Grains	−406 (−863 to +50)	+3 (−210 to +217)	+410 (−81 to +900)	0.10	−85 (−372 to +203)	−224 (−588 to +140)	−139 (−605 to +326)	0.54	0.10
- Whole grains	+57 (−47 to +160)	+49 (−38 to +135)	−8 (−162 to +145)	0.91	+80 (−17 to +176)	+14 (−117 to +146)	−65 (−211 to +80)	0.36	0.58
- Some whole grains	−60 (−182 to +63)	+60 (−90 to +209)	+119 (−72 to +311)	0.21	+11 (−15 to +37)	−6 (−85 to +73)	−17 (−98 to +63)	0.66	0.18
- Refined grains	−403 (−832 to +26)	−105 (−342 to +132)	+298 (−171 to +767)	0.20	−175 (−456 to +106)	−232 (−594 to +130)	−57 (−540 to +427)	0.81	0.28
Meat	−1,435 (−3,361 to +492)	−4,912 (−6,475 to −3,349)***	−3,478 (−6,096 to −859)	0.01	+22 (−1,781 to +1,825)	−4,882 (−6,097 to −3,667)***	−4,904 (−7,239 to −2,569)	<0.001	0.40
- Red meat	−1,140 (−2,066 to −214)*	−595 (−1,139 to −51)*	+545 (−525 to +1,615)	0.30	−445 (−799 to −91)*	−1,426 (−2,187 to −664)***	−981 (−1,717 to −244)	0.01	0.02
- White meat	−257 (−1,956 to +1,443)	−4,075 (−5,498 to −2,652)***	−3,818 (−6,201 to −1,436)	0.003	+500 (−1,186 to +2,186)	−2,976 (−3,898 to −2,054)***	−3,476 (−5,442 to −1,511)	0.001	0.82
- Fried meat	−37 (−335 to +260)	−913 (−2,043 to +218)	−875 (−2,050 to +299)	0.14	−78 (−562 to +406)	−412 (−956 to +132)	−334 (−1,070 to +402)	0.36	0.43
- Processed meat	−38 (−325 to +249)	−242 (−473 to −11)*	−204 (−537 to +129)	0.22	−33 (−167 to +101)	−480 (−719 to −241)***	−447 (−704 to −190)	0.001	0.24
- Seafood	+444 (+42 to +847)*	−592 (−921 to −263)**	−1,036 (−1,569 to −503)	<0.001	+469 (−146 to +1,084)	−822 (−1,293 to −350)**	−1,291 (−2,058 to −524)	0.002	0.58
Eggs	−34 (−519 to +451)	−539 (−840 to −238)**	−505 (−0 to −1,009)	0.05	+251 (−59 to +561)	−519 (−840 to −197)**	−797 (−1,188 to −351)	<0.001	0.41
- Baked	−6 (−15 to +3)	−3 (−7 to +1)	+3 (−7 to +12)	0.52	−5 (−10 to +1)	−10 (−17 to −3)**	−6 (−13 to +2)	0.17	0.16
- Boiled	−0 (−4 to +3)	−1 (−3 to +1)	−1 (−5 to +3)	0.72	+2 (−3 to +6)	+0 (0 to 0)	−2 (−6 to +3)	0.41	0.72
- Fried	−27 (−509 to +454)	−534 (−835 to −235)**	−507 (−1,008 to −6)	0.047	+254 (−58 to +566)	−509 (−831 to −186)**	−762 (−1,180 to −345)	<0.001	0.42
Nuts & Seeds	+65 (−534 to +664)	−70 (−483 to +344)	−134 (−957 to +688)	0.74	+891 (+402 to +1,380)***	−753 (−1,225 to −282)	−1,644 (−2,283 to −1,005)	<0.001	0.004
Meat alternatives	+35 (−68 to +138)	+482 (+213 to +751)**	+447 (+225 to +669)***	<0.001	+95 (−281 to +472)	+473 (+179 to +767)**	+378 (−53 to +809)	0.08	0.77
Dairy	−1,550 (−3,323 to +224)	−1,668 (−2,706 to −631)**	−119 (−2,285 to +2,048)	0.91	−367 (−1,551 to +817)	−2,934 (−4,161 to −1,708)***	−2,568 (−3,605 to −1,530)***	<0.001	0.04
- Full-fat	−1,436 (−2,995 to +124)	+305 (−137 to +746)	+1,741 (+51 to +3,431)	0.04	+566 (+216 to +916)**	−2,535 (−3,766 to −1,304)	−3,101 (−4,376 to −1,826)	<0.001	<0.001
- Reduced-fat	−129 (−533 to +276)	−35 (−94 to +23)	+93 (−280 to +466)	0.61	+216 (−71 to +503)	−393 (−631 to −155)**	−609 (−1,086 to −132)	0.01	0.02
- Non-fat	+15 (−20 to +50)	−1,938 (−2,865 to −1,010)***	−1,953 (−2,885 to −1,020)	<0.001	−1,149 (−2,231 to −66)*	−6 (−16 to +4)	+1,142 (+59 to +2,226)	0.04	<0.001
Dairy alternatives	+2 (−3 to +8)	−481 (−811 to −152)**	−484 (−813 to −154)	0.006	−174 (−361 to +13)	+7 (−1 to +15)	+181 (−8 to +370)	0.06	<0.001
Added fat	+1,578 (+588 to +2,267)**	−900 (−1,488 to −312)**	−2,477 (−3,724 to −1,231)	<0.001	+1,844 (+1,080 to +2,608)***	−1,230 (−1,709 to −750)***	−3,074 (−3,886 to −2,262)	<0.001	0.40
- Animal	−711 (−1,354 to −69)*	−276 (−574 to +22)	+435 (−161 to +1,031)	0.14	−264 (−585 to +56)	−914 (−1,266 to −563)***	−650 (−1,081 to −219)	0.005	0.003
- Vegetable	+2,289 (+1,423 to +3,154)***	−624 (−1,118 to −130)*	−2,912 (−4,144 to −1,681)	<0.001	+2,109 (+1,329 to +2,888)***	−315 (−565 to −66)*	−2,424 (−3,228 to −1,620)	<0.001	0.49
Added sugar	−32 (−83 to +20)	+2 (−93 to +98)	+34 (−65 to +134)	0.49	−7 (−139 to +124)	−58 (−124 to +8)	−51 (−194 to +92)	0.47	0.32
Sugar-sweetened beverages	−1 (−3 to +0)	−1 (−2 to −0)*	+0 (−1 to +2)	0.65	−1 (−2 to +0)	−2 (−3 to −0)*	−1 (−2 to +0)	0.11	0.19
Alcohol	+1 (−7 to +8)	−3 (−8 to +1)	−4 (−11 to +3)	0.25	+2 (−3 to +7)	−4 (−8 to +1)	−6 (−13 to +1)	0.08	0.64

Data are means and estimated treatment effects with 95% confidence intervals. *P* values for treatment effect are from paired *t*-tests comparing mean changes within each participant while on each treatment arm. *P*-values for carryover effect are from two-sample *t*-tests comparing treatment effects between patients whose first diet was Mediterranean versus vegan.

*p < 0.05,

**p < 0.01, and

***p < 0.001 for within-group changes from baseline assessed by paired *t*-tests.

**TABLE 3:** Changes in dietary Advanced Glycation End-Products (AGEs), comparing a Mediterranean and low-fat vegan diet, in the first 16 weeks of the study.

Variable	Mediterranean Baseline	Mediterranean Final	Δ Mediterranean	Vegan Baseline	Vegan Final	Δ Vegan	Treatment Effect	P-value (t test)
**Dietary AGEs (ku/day)**								
Total AGEs (ku/day)	13,022 (10,554–15,489)	12,009 (10.197–13,820)	−1,013 (−3,946 to +1,920)	13,469 (11,436–15,503)	2,878 (2,340–3,416)	−10,591 (−12,499 to −8,683)***	−9,568 (−13,014 to −6,143)	<0.001
Fruit	162 (22–301)	179 (44–313)	+17 (−141 to +175)	311 (97–525)	30 (0–70)	−281 (−499 to −63)*	−298 (−558 to −38)	0.03
Vegetables	172 (119–224)	203 (152–254)	+32 (−47 to +110)	196 (119–273)	271 (210–333)	+76 (−14 to +166)	+45 (−72 to +161)	0.45
Grains	860 (543–1,177)	459 (331–587)	−400 (−755 to −45)*	952 (638–1,267)	787 (572–1,003)	−165 (−516 to +186)	+235 (−255 to +725)	0.34
- Whole grains	148 (87–210)	193 (122–263)	+44 (−48 to +136)	239 (135–343)	245 (168–323)	+6 (−117 to +129)	−38 (−186 to +111)	0.61
- Some whole grains	91 (15–166)	42 (0 to 94)	−49 (−143 to +45)	50 (0–110)	55 (12–97)	+5 (−72 to +81)	+53 (−66 to +173)	0.37
- Refined grains	621 (326–915)	225 (118–332)	−395 (−724 to −66)*	663 (417–909)	487 (265–710)	−176 (−525 to +173)	+220 (−250 to +689)	0.35
Meat	4,917 (3,599–6,235)	3,683 (2,746–4,620)	−1,234 (−2,785 to +316)	4,725 (3,575–5,876)	20 (0–48)	−4,705 (−5,860 to 3,552)***	−3,471 (−5,397 to −1,546)	<0.001
-Red meat	1,557 (883–2,232)	519 (106–932)	−1,038 (−1,824 to −252)*	1,445 (747–2,143)	20 (0–48)	−1,426 (−2,129 to −722)***	−388 (−1,429 to +653)	0.46
- White meat	3,023 (1,957–4,088)	2,900 (2,021–3,779)	−123 (−1,471 to +1,226)	2,835 (1,953–3,716)	0 (0–0)	−2,835 (−3,716 to −1,953)	−2,712 (−4,294 to −1,130)	0.001
- Fried meat	258 (0–530)	92 (0–224)	−167 (−480 to +146)	383 (0–887)	0 (0–0)	−383 (−887 to +122)	−216 (−799 to +367)	0.46
- Processed meat	330 (156–505)	255 (97–413)	−75 (−329 to +179)	446 (219–672)	0 (0–0)	−446 (−672 to −219)***	−371 (−706 to −35)	0.03
- Seafood	648 (372–924)	1,154 (837–1,471)	+507 (+154 to +859)**	821 (381–1,262)	13 (0–32)	−808 (−1,249 to −367)***	−1,314 (−1,862 to −767)	<0.001
Eggs	616 (296–935)	571 (329–812)	−45 (−433 to +343)	515 (214–816)	0 (0–1)	−515 (−816 to −214)**	−470 (−958 to +18)	0.06
- Baked	9 (3–15)	6 (2–10)	−3 (−11 to +5)	10 (4–17)	0 (0–1)	−10 (−16 to −4)**	−7 (−17 to +3)	0.18
- Boiled	5 (1–9)	3 (0–6)	−2 (−7 to +3)	0 (0–0)	0 (0–0)	0 (0–0)	+2 (−3 to +7)	0.46
- Fried	602 (285–918)	562 (321–802)	−40 (−424 to +345)	505 (203–807)	0 (0–0)	−505 (−807 to −203)**	−465 (−951 to +21)	0.06
Nuts & Seeds	908 (382–1,435)	1,076 (636–1,516)	+167 (−313 to +648)	992 (604–1,379)	233 (75–391)	−759 (−1,197 to −320)**	−926 (−1,567 to −285)	0.005
Meat alternatives	19 (0–58)	46 (0–112)	+26 (−52 to +105)	99 (0–225)	547 (321–773)	+448 (+173 to +722)**	+421 (+138 to +705)	0.005
Dairy	2,637 (1,290–3,983)	1,161 (647–1,675)	−1,476 (−2,918 to −33)*	2,992 (1,825–4,158)	64 (0–176)	−2,927 (−4,092 to −1,763)***	−1,452 (−3,291 to +387)	0.12
- Full-fat	2,212 (1,024–3,399)	955 (475–1,435)	−1,257 (−2,533 to +20)	2,567 (1,394–3,740)	21 (0–48)	−2,546 (−3,718 to −1,374)***	−1,289 (−2,998 to +419)	0.14
- Reduced-fat	423 (118–728)	193 (42–343)	−231 (−591 to +130)	418 (202–634)	43 (0–132)	−375 (−597 to −153)**	−145 (−561 to +271)	0.49
- Non-fat	2 (0–4)	14 (0–40)	+12 (−15 to +38)	6 (0–15)	0 (0–0)	−6 (−15 to +3)	−17 (−45 to +10)	0.21
Dairy alternatives	2 (0–5)	4 (0–11)	+2 (−2 to +6)	2 (0–4)	9 (1–16)	+7 (−1 to +14)	+5 (−4 to +13)	0.28
Added fat	1,970 (1,350–2,589)	3,368 (2,727–4,009)	+1,399 (+567 to +2,231)**	1,658 (1,230–2,086)	312 (192–432)	−1,346 (−1,820 to −873)***	−2,745 (−3,686 to −1,803)	<0.001
-Animal	1,172 (585–1,760)	358 (107–610)	−814 (−1,423 to −205)*	972 (621–1,324)	28 (11–44)	−945 (−1,298 to −592)***	−131 (−823 to +562)	0.71
- Vegetable	797 (561–1,034)	3,010 (2,314–3,706)	+2,213 (+1,534 to +2,892)***	686 (432–940)	285 (171–399)	−401 (−700 to −102)*	−2,614 (−3,347 to −1,881)	<0.001
Added sugar	59 (18–99)	27 (0–58)	−32 (−74 to +10)	65 (6–125)	6 (0–17)	−59 (−120 to +3)	−26 (−97 to +45)	0.46
Sugar-sweetened beverages	3 (1–4)	1 (0–3)	−1 (−3 to −0)*	2 (1–4)	1 (0–1)	−2 (−3 to −0)*	−0 (−2 to +2)	0.72
Alcohol	7 (1–12)	7 (3–12)	+1 (−5 to +7)	9 (4–14)	6 (2–9)	−4 (−8 to +1)	−4 (−11 to +3)	0.23

Data shown are means and estimated treatment effects with 95% confidence intervals. The treatment effect is the mean (average) difference between participant outcomes on the vegan versus the Mediterranean diet. *P*-values for treatment effect are from two-sample *t*-tests comparing mean changes between patients on Mediterranean diet and patients on vegan diet during the first 16 weeks of the study.

*p < 0.05,

**p < 0.01 and

***p < 0.001 for paired comparison t-tests assessing whether within-group changes from baseline are different from zero.

### Body weight and body composition

**FIGURE 2:**
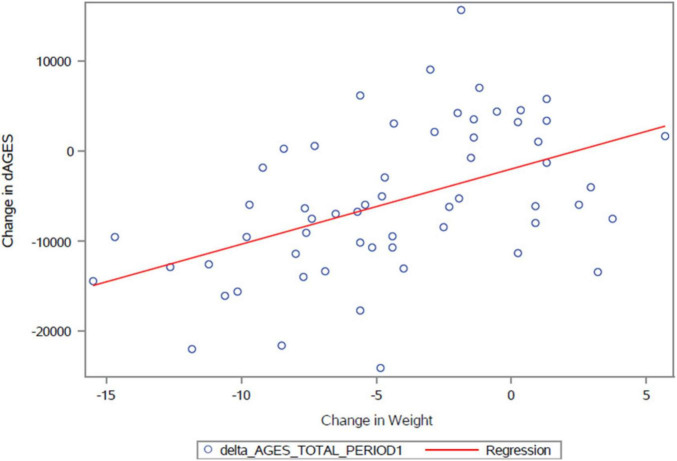
Relationship between changes in dietary AGEs and changes in body weight in the first study period; *r* = +0.48; *p* < 0.001.

As reported earlier (9), the participants lost 6.0 kg on average on the vegan diet, compared with no change on the Mediterranean diet (treatment effect −6.0 kg [95% CI −7.5 to −4.5]; *p* < 0.001). Most of the weight loss on the vegan diet was attributable to a reduction in fat mass (treatment effect −3.4 kg [95% CI −4.7 to −2.2]; *p* < 0.001) and visceral fat volume (treatment effect −314.5 cm^3^ [95% CI −446.7 to −182.4]; *p* < 0.001). Changes in dietary AGEs correlated with changes in body weight (*r* = +0.48; *p* < 0.001 for period 1; Figure 2) and remained significant after adjustment for total energy intake (*r* = +0.39; *p* = 0.003).

## Discussion

This 36-week crossover trial, which compared a Mediterranean and a low-fat vegan diet head-to-head, demonstrated a 73% reduction in dietary AGEs on the low-fat vegan diet, compared with no change on the Mediterranean diet. Changes in AGEs strongly correlated with weight changes, independent of energy intake.

The Mediterranean diet has been previously shown to reduce dietary and serum AGEs in elderly adults, compared with a Western diet rich in saturated fat (16). In contrast to the experimental Western diet used in this study, which contained 22% energy in the form of saturated fat, our study population was more health-conscious at baseline and consumed only 10% energy from saturated fat, which may have explained no observed reduction in dietary AGEs on the Mediterranean diet in the current study. A separate study of a Mediterranean diet in people with type 2 diabetes with previous cardiovascular events also demonstrated a reduction in serum AGEs, compared with a control low-fat diet, and both of these diets reduced the dietary AGEs (17). The low-fat diet in this study contained animal products and was higher in fat (30% energy) compared with our study (17% energy), which may have played a role in a less-expressed reduction in the dietary AGEs compared with the current study. The reduction in dietary AGEs with a low-fat vegan diet has been reported previously in overweight adults (15) and in postmenopausal women (18), in both cases compared with a control (habitual) diet. This study, for the first time, compares the effects with a Mediterranean diet to a low-fat vegan diet, and shows a beneficial effect of a low-fat vegan diet.

This study also elucidated which dietary changes contributed most to the reduction of dietary AGEs on the low-fat vegan diet, namely excluding the consumption of meat, minimizing added fat, and avoiding dairy products. These findings are supported by the relatively low AGE content in plant foods, compared with animal-derived and high-fat foods (5). Randomized interventional trials have shown that modifying cooking methods so as to reduce dietary AGEs ameliorates insulin resistance in obese people with metabolic syndrome (6, 7). A low-fat vegan diet achieves a significant reduction in dietary AGEs through qualitative dietary changes, without changing food preparation techniques (15). Plant-based diets have been shown to reduce the risk of developing the metabolic syndrome and type 2 diabetes by about one half (19, 20), and the lower intake of AGEs on these diets may partly explain their benefits.

AGEs can contribute to the pathogenesis of insulin resistance and increased body weight through several mechanisms (21), including (1) a direct modification of signaling molecules, such as insulin itself, which reduces its biological activity (22) and affinity for the insulin receptor, hereby impairing insulin signaling, or the modification of the three arginine residues in the AMP binding site of AMP kinases, decreasing their activity (23); and (2) interference with activation of downstream proteins involved in cell insulin signaling, including IRS-1 and Akt, through RAGE-dependent induction of proinflammatory cytokines and reactive oxygen species (24).

The current trial did not observe any effect of a Mediterranean diet on weight loss, which is in line with previous studies that did not include energy restriction or exercise. The Lyon Diet Heart Study (25) used a Mediterranean diet supplemented with margarine enriched in an alpha-linolenic acid in studying secondary prevention of cardiovascular disease, but found a small weight gain (1.4 kg) during the 2-year study (26). In the PREDIMED study, which included mostly overweight people (27), the weight loss in the first 3 months was very small (0.19 kg in the olive oil-supplemented group and 0.26 kg in the nut-supplemented group) (10).

A 2020 large prospective study that included more than 255,000 adults from ten European countries showed that increased consumption of dietary AGEs was associated with additional weight gain over the 5-year follow-up period (28). Increased AGEs have also been associated with markers of insulin resistance and inflammation, and the risk of developing the metabolic syndrome (29). Some studies suggest that accumulation of AGEs in the extracellular matrix plays a key role in the development of obesity-related adipose tissue dysfunction (30, 31), and the reduction in their intake may therefore be an effective strategy to counteract these negative consequences.

This study has several strengths. The cross-over design enabled the research team to directly compare the effects of a Mediterranean and a low-fat diet. The reasonably high retention (84%) suggests that both diets are doable and acceptable. The study duration was long enough to allow sufficient time for metabolic adaptation (32). Because this was a free-living study, the results can readily translate to nonclinical settings. The study also has limitations. As we examined a modestly large number of outcomes, there is a possibility of some false positive findings due to multiple comparisons. However, the treatment effects reported for total dietary AGEs and several other outcomes, with unadjusted *p*-values < 0.001, remain significant at the 0.05 level after conservative (Bonferroni) correction for the total number of outcomes examined. Although self-reported overall adherence to both diets was high, the limitations of self-reported dietary intake are well-known. The detected carry-over effect for total dietary AGEs necessitated more detailed statistical analyses, including a conservative estimate from an ANOVA for repeated measures from the first 16 weeks of the study alone; findings from this alternate analysis were generally very consistent with the full crossover model.

## Conclusion

This study suggests that a low-fat vegan diet is an effective strategy for reduction in dietary AGEs and for weight loss, compared with a Mediterranean diet. Further studies are needed to confirm these findings.

## Data availability statement

The raw data supporting the conclusions of this article will be made available by the authors, without undue reservation.

## Ethics statement

The studies involving humans were approved by the Advarra Institutional Review Board, located in Columbia, MD, USA. The studies were conducted in accordance with the local legislation and institutional requirements. The participants provided their written informed consent to participate in this study.

## Author contributions

HK: Conceptualization, Investigation, Methodology, Supervision, Writing – original draft. TZ-M: Data curation, Methodology, Project administration, Writing – review and editing. GM: Data curation, Project administration, Writing – review and editing. EE: Project administration, Writing – review and editing. AP: Project administration, Writing – review and editing. JU: Methodology, Project administration, Supervision, Writing – review and editing. RH: Formal analysis, Writing – review and editing. NB: Funding acquisition, Methodology, Supervision, Writing – review and editing.
