# Supplementary material for: Dietary advanced glycation end-products and their associations with body weight on a Mediterranean diet and low-fat vegan diet: a randomized, cross-over trial
Source: Front Nutr. 2024 Aug 8;11:1426642. doi: 10.3389/fnut.2024.1426642 (PMC11340516; doi:10.3389/fnut.2024.1426642)
Supplement: Supplementary file 1 [file Table_1.docx]

**Table 1. Baseline Characteristics of the Study Population.**

| **Characteristic** | **Group 1**  **(n=32)** | **Group 2**  **(n=30)** |  | **P-Value** |
| --- | --- | --- | --- | --- |
| **Age (years)** | 56.6 | 58.3 |  | 0.50 |
| **Sex (number, %)** |  |  |  |  |
| Female | 26 (81.3) | 22 (73.3) |  | 0.46 |
| Male | 6 (18.8) | 8 (26.7) |  |  |
| **Race, (number, %)**^1^ |  |  |  |  |
| White | 15 (46.9) | 16 (53.3) |  | 0.90 |
| Black | 16 (50.0) | 14 (46.7) |  |  |
| Asian, Pacific Islander | 0 (0.0) | 0 (0.0) |  |  |
| American Indian, Eskimo, Aleut | 1 (3.1) | 0 (0.0) |  |  |
| Not disclosed | 0 (0.0) | 0 (0.0) |  |  |
| **Ethnicity, (number, %)**^1^ |  |  |  |  |
| Non-Hispanic | 23 (71.9) | 23 (76.7) |  | 0.14 |
| Hispanic | 3 (9.4) | 0 (0.0) |  |  |
| Not disclosed | 6 (18.8) | 7 (23.3) |  |  |
| **Marital status** |  |  |  |  |
| Not married | 15 (46.9) | 15 (50.0) |  | 0.71 |
| Married | 17 (53.1) | 14 (46.7) |  |  |
| Not disclosed | 0 (0.0) | 1 (3.3) |  |  |
| **Education** |  |  |  |  |
| High school | 0 (0.0) | 0 (0.0) |  | 0.28 |
| Associates | 7 (21.9) | 5 (16.7) |  |  |
| College | 13 (40.6) | 9 (30.0) |  |  |
| Graduate degree | 12 (37.5) | 16 (53.3) |  |  |
| **Occupation** |  |  |  |  |
| Service occupation | 7 (21.9) | 4 (13.3) |  | 0.29 |
| Technical, sales, administrative | 8 (25.0) | 9 (30.0) |  |  |
| Professional or managerial | 2 (6.3) | 7 (23.3) |  |  |
| Retired | 7 (21.9) | 6 (20.0) |  |  |
| Other | 8 (25.0) | 4 (13.3) |  |  |
| **Medications** |  |  |  |  |
| Lipid-lowering therapy (%) | 12 (37.5) | 11 (36.7) |  | 0.95 |
| Antihypertensive therapy (%) | 16 (50.0) | 14 (46.7) |  | 0.79 |
| Thyroid medications (%) | 3 (9.4) | 1 (3.3) |  | 0.61 |
| **Physical Activity (METs)** | 2289.7 | 2665.5 |  | 0.70 |
| **Energy intake (kcals)** | 1825.8 | 1911.8 |  | 0.54 |
| **Anthropometrics** |  |  |  |  |
| Body weight (kg) | 97.6 | 98.4 |  | 0.80 |
| BMI (kg/m^2^) | 34.3 | 33.7 |  | 0.42 |
| Fat mass (g) | 43.9 | 41.5 |  | 0.17 |
| Lean mass (g) | 51.5 | 54.1 |  | 0.25 |
| VAT volume (cm^3^)^2^ | 2017.4 | 2126.7 |  | 0.68 |
| **Lipids** |  |  |  |  |
| Total cholesterol (mg/dL) | 203.3 | 202.2 |  | 0.93 |
| LDL-cholesterol (mg/dL) | 119.9 | 116.6 |  | 0.76 |
| HDL-cholesterol (mg/dL) | 58.8 | 56.4 |  | 0.53 |
| **HbA1c** | 5.8 | 5.8 |  | 0.93 |
|  |  |  |  |  |

Data are means ± SD, or number (%). P-values refer to t-tests for continuous variables and χ*^2^* or Fisher’s exact test for categorical variables. The P-value calculated for ethnicity distribution is for the comparison between Hispanic vs. non-Hispanic categories and all other comparisons also exclude undisclosed datapoints. Group 1 started with the Mediterranean diet and Group 2 started with the vegan diet. ^1^Race and ethnicity were determined via self-report.

^2^VAT: visceral adipose tissue
