# Supplementary figures and images for: Dietary advanced glycation end-products and their associations with body weight on a Mediterranean diet and low-fat vegan diet: a randomized, cross-over trial
Source: Front Nutr. 2024 Aug 8;11:1426642. doi: 10.3389/fnut.2024.1426642 (PMC11340516; doi:10.3389/fnut.2024.1426642)

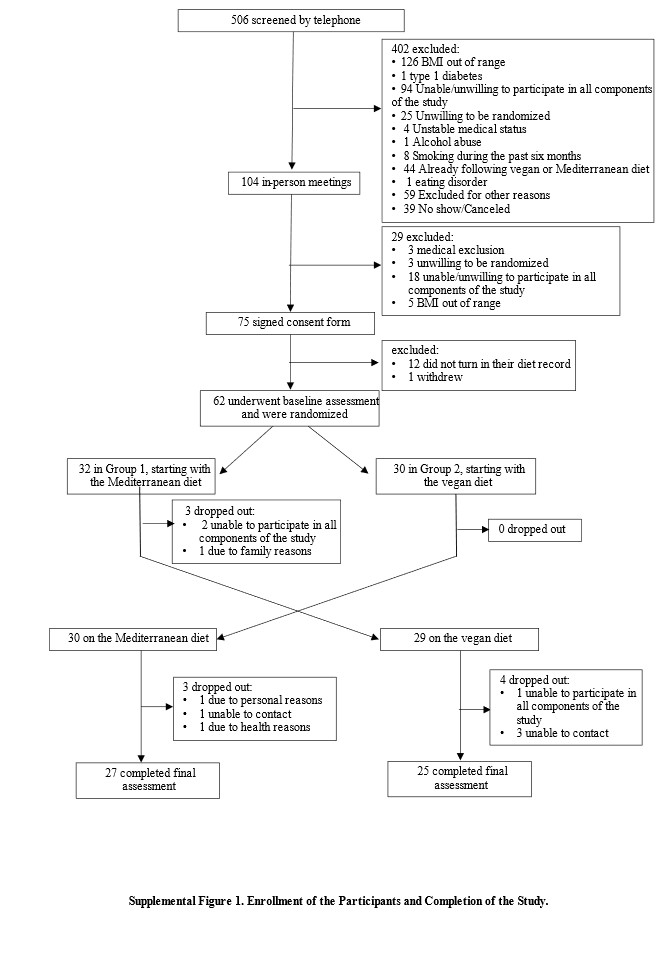

Supplement: Supplementary file 2 [file Image_1.jpeg]
